# Impact of Molecular Epidemiology and Reduced Susceptibility to Glycopeptides and Daptomycin on Outcomes of Patients with Methicillin-Resistant *Staphylococcus aureus* Bacteremia

**DOI:** 10.1371/journal.pone.0136171

**Published:** 2015-08-21

**Authors:** Hao-Yuan Lee, Chyi-Liang Chen, Shu-Ying Liu, Yu-Shan Yan, Chee-Jen Chang, Cheng-Hsun Chiu

**Affiliations:** 1 Graduate Institute of Clinical Medical Sciences, Chang Gung University College of Medicine, Taoyuan, Taiwan; 2 Department of Pediatrics, Chang Gung Children’s Hospital, Chang Gung University College of Medicine, Taoyuan, Taiwan; 3 Molecular Infectious Disease Research Center, Chang Gung Memorial Hospital, Chang Gung University College of Medicine, Taoyuan, Taiwan; 4 Department of Molecular Biotechnology, Da-Yeh University, Changhua, Taiwan; Institut National de la Recherche Agronomique, FRANCE

## Abstract

**Background:**

Methicillin-resistant *Staphylococcus aureus* (MRSA) bacteremia was associated with high mortality, but the risk factors associated with mortality remain controversial.

**Methods:**

A retrospective cohort study was designed. All patients with MRSA bacteremia admitted were screened and collected for their clinical presentations and laboratory characteristics. Minimum inhibitory concentration (MIC) and staphylococcal cassette chromosome *mec* (SCC*mec*) type of bacterial isolates were determined. Risk factors for mortality were analyzed.

**Results:**

Most MRSA isolates from the 189 enrolled patients showed reduced susceptibility to antibiotics, including MIC of vancomycin ≥ 1.5 mg/L (79.9%), teicoplanin ≥ 2 mg/L (86.2%), daptomycin ≥ 0.38 mg/L (73.0%) and linezolid ≥ 1.5 mg/L (64.0%). MRSA with vancomycin MIC ≥ 1.5 mg/L and inappropriate initial therapy were the two most important risk factors for mortality (both *P* < 0.05; odds ratio = 7.88 and 6.78). Hospital-associated MRSA (HA-MRSA), carrying SCC*mec* type I, II, or III, was associated with reduced susceptibility to vancomycin, teicoplanin or daptomycin and also with higher attributable mortality (all *P* < 0.05). Creeping vancomycin MIC was linked to higher MIC of teicoplanin and daptomycin (both *P* < 0.001), but not linezolid (*P* = 0.759).

**Conclusions:**

Giving empirical broad-spectrum antibiotics for at least 5 days to treat catheter-related infections, pneumonia, soft tissue infection and other infections was the most important risk factor for acquiring subsequent HA-MRSA infection. Choice of effective anti-MRSA agents for treating MRSA bacteremia should be based on MIC of vancomycin, teicoplanin and daptomycin. Initiation of an effective anti-MRSA agent without elevated MIC in 2 days is crucial for reducing mortality.

## Introduction

*Staphylococcus aureus* bacteremia was associated with high risk of mortality, longer hospital stay, and increased costs for patients and the health care system than bacteraemia due to other bacterial pathogens [1,2]. Vancomycin is still the drug widely used for treating methicillin-resistant *S*. *aureus* (MRSA) infections, but several studies have reported a creeping tendency for vancomycin minimum inhibitory concentration (MIC) within the susceptible range (≤ 2 mg/L) [3]. Clinical treatment failure was found in association with creeping vancomycin MIC in some studies, but others showed no correlation [4, 5]. These findings should be considered when interpreting vancomycin susceptibility and in determining whether alternative antistaphylococcal agents are necessary for patients infected by MRSA with elevated but susceptible vancomycin MIC values. Furthermore, a higher teicoplanin MIC (> 1.5 mg/L) has been proved to predict an unfavourable outcome and higher mortality rate among teicoplanin-treated MRSA bacteraemic patients [6]. However, there are no studies to assess whether the outcome is associated with MICs of other alternative antistaphylococcal agents, such as daptomycin and linezolid.

The molecular classification system of MRSA is based on the staphylococcal cassette chromosome *mec* (SCC*mec*) [7]. The association of molecular epidemiology of SCC*mec* types with vancomycin MIC was found in previous studies [8–10]. A significant association between SCC*mec* II and elevated vancomycin MIC was reported before [8]. The higher vancomycin MIC was associated with specific clonal complexes (CCs) and hospital-associated MRSA, as described in previous studies [10, 11]. Risk factors associated with mortality, such as SCC*mec* types, inappropriate initial therapy, MIC of antistaphylococcal agents, hospital- or community-associated MRSA, and disease severity were reported in earlier studies but remained incomplete and controversial [12, 13]. The present study tried to fill the gap by applying backward root analysis to survey, step by step, the independent risk factors associated with mortality due to MRSA bacteremia. We analyzed clinical characteristics, microbiological features, and final outcomes of bacteremic patients stratified by both SCC*mec* types and the antimicrbial MICs of the isolates.

## Materials and Methods

### Ethics statement and inclusion criteria

This study was approved by the Institutional Review Board of the Chang Gung Memorial Hospital which waived the requirement of informed consent (approval reference number 100-3588B). Medical records were reviewed for all admitted patients with ≥ 1 positive blood culture for *S*. *aureus* and symptoms and signs of infection. For patients with multiple episodes of bacteremia, only the first episode was included. Patients with incomplete medical records, younger than 18 years of age, or with polymicrobial infection were excluded.

### Antimicrobial susceptibility testing and vancomycin-heteroresistant *S*. *aureus* screening

The MICs of vancomycin, daptomycin, teicoplanin, and linezolid were determined by the Etest (AB Biodisk, Solna, Sweden) [14]. The interpretation was based on Clinical and Laboratory Standards Institute (CLSI) standards [15]. Breakpoints for vancomycin, teicoplanin, daptomycin and linezolid were summarized as shown in supplemental information (S1 Table). Screening for vancomycin-heteroresistant *S*. *aureus* (hVISA) was performed by the vancomycin-teicoplanin Etest macromethod, according to the manufacturer’s instructions (AB Biodisk). ATCC29213 was used as a control in the MIC and hVISA screening tests. An isolate was considered hVISA when growing in the presence of teicoplanin ≥ 12 μg alone or ≥ 8 μg for both vancomycin and teicoplanin [15].

### Staphylococcal cassette chromosome *mec* typing

The staphylococcal cassette chromosome *mec* (SCC*mec*) types of all 189 isolates were determined by a multiplex polymerase chain reaction (PCR) described previously [16], and another multiplex PCR strategy was used if this method was not successful in identifying a type [17]. The identification of SCC*mec* type V_T_ was verified with a method described by Boyle-Vavra et al [18].

### Genotyping

Multilocus sequence typing (MLST) based on seven housekeeping genes was performed on all isolates [19]. Panton-Valentine leucocidin (PVL) genes (*lukF-PV* and *lukS-PV*) were checked by PCR—based assays as previously described [20].

### Clinical data collection and definitions

Clinical data were collected retrospectively. Mortality was defined as bacteremia-attributable death, i.e., death before resolution of symptoms and signs of bacteremia and at least a blood culture positive for MRSA [21]. Considering “time at risk” for bacteria to become resistant to antimicrobial agents under antimicrobial selective pressure, prior exposure to these agents was defined as at least 5 days of therapy during the 14 days before the isolation of MRSA [21, 22]. Appropriate initial antimicrobial therapy was defined as giving patients with at least one susceptible antimicrobial agent, except single aminoglycoside or rifampicin treatment, within 2 days after the onset of bacteremia [12]. Multidrug resistance (MDR) was defined as resistance to at least 3 antibiotic classes [21]. Culture detecting time was the interval (days) from culture sampling to reporting [21]. Community-associated MRSA (CA-MRSA) was defined as an isolate possessing the SCC*mec* type IV or V genes, and hospital-associated MRSA (HA-MRSA) was defined as an isolate carrying SCC*mec* type I, II, or III genes [11]. Community-acquired infection (CAI) was defined as the isolation of MRSA from bloodstream within 48 h of admission, while hospital-acquired infection (HAI) as beyond that time [23]. Catheter-related infection was defined by the evidence of infected intravascular catheter which was considered to be the portal of entry if a catheter-tip culture was positive for *S*. *aureus* or if inflammation was present around the catheter insertion site.

### Multi-stages risk factor analysis

An analytical model with multiple stages was proposed from the appearance of MRSA infection to mortality for patients with mortality [21]. The most important main risk factor (MRF1) associated with mortality due to MRSA infection was the first one to be analyzed by multivariate logistic analysis. Then main risk factor (MRF2) correlated with MRF1 was the second one to be analyzed by the same method. In this way, MRF1, MRF2, MRF3, MRF4, etc. would be identified as the most important independent risk factor in each stage backward from death to infection [21].

### Statistical analysis

Data were recorded and entered into a database. Analyses were performed using SPSS software, v. 17.0 (SPSS Inc., Chicago, IL, USA). The Student’s t-test, the Chi-square test, Fisher’s exact test or ANOVA was used when appropriate to compare proportions. Variables with a *P* value < 0.2 in the univariate analysis were added in a stepwise manner and selected to determine the final model for multivariable analysis. All statistical analyses were two-sided, and significance was set at *P* < 0.05.

## Results

### Bacterial isolates and patients

A total of 501 isolates among patients with *S*. *aureus* bacteraemia treated in Chang Gung Memorial Hospital from January 2010 to December 2011 were collected. A total of 252 MRSA isolates was collected and the resistance rate to oxacillin among *S*. *aureus* was 50.3%. After review, 63 patients were excluded including 4 patients < 18 years old, 32 with polymicrobial infection, 25 non-first, repetitive episodes, and 2 with insufficient medical records. A total of 189 patients with symptoms and isolation of MRSA from bloodstream met the inclusion criteria.

### Reduced susceptibility to anti-MRSA agents

**Fig 1:**
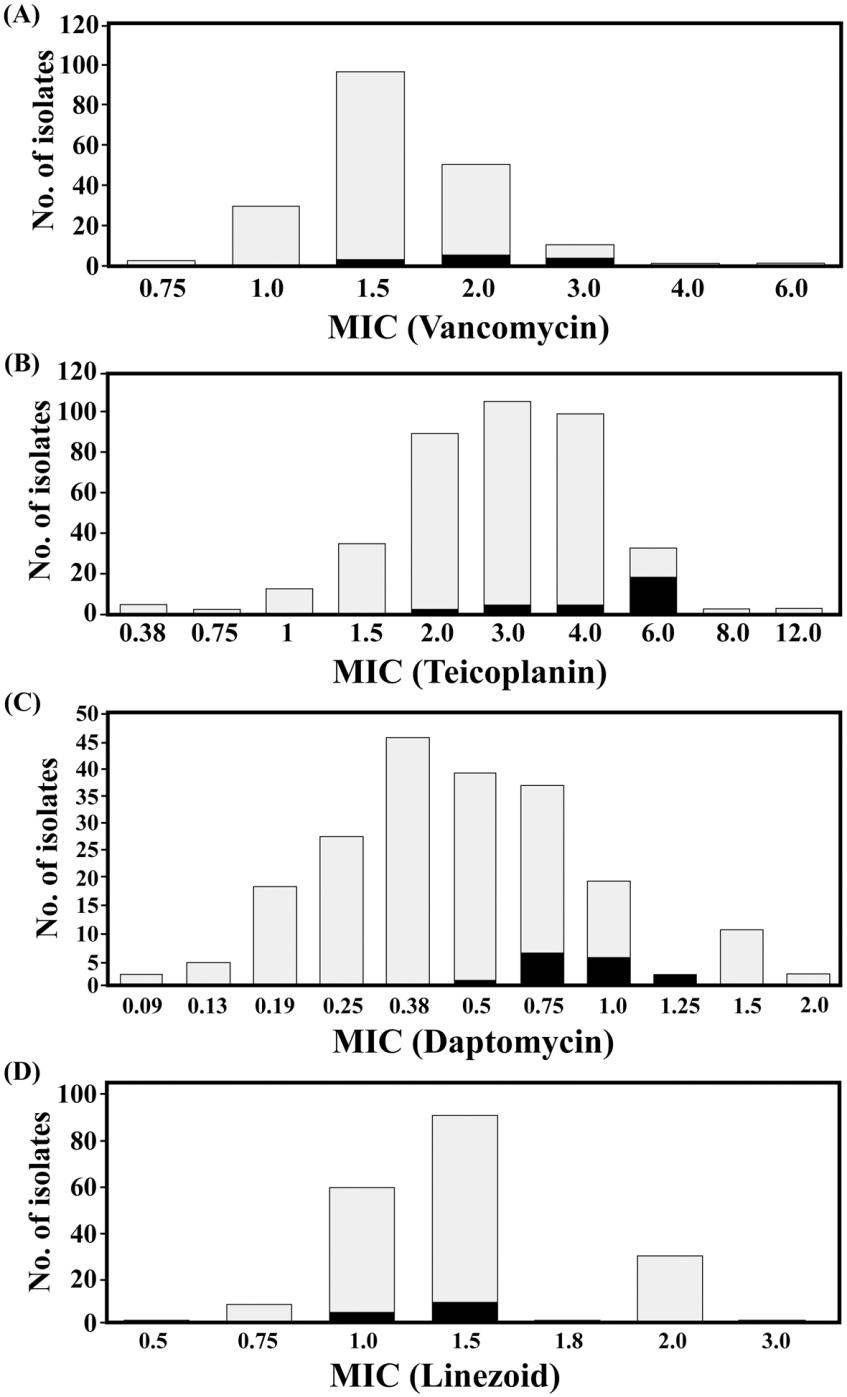
Distribution of methicillin-resistant *Staphylococcus aureus* (MRSA) bloodstream isolates with different minimum inhibitory concentrations (MICs) (mg/L) of various anti-MRSA agents (A, vancomycin; B, teicoplanin; C, daptomycin; D, linezolid). The isolate number of hVISA is shown in black.

Using CLSI breakpoints as shown in supplemental information (S1 Table), we found reduced susceptibilities in vancomycin, teicoplanin, daptomycin and linezolid among the isolates (Fig 1). Most isolates showed vancomycin MIC ≥ 1.5 mg/L (79.9%), teicoplanin MIC ≥2 mg/L (86.2%), daptomycin MIC ≥ 0.38 mg/L (73.0%) and linezolid MIC ≥ 1.5 mg/L (64.0%) (Table 1). By linear regression analysis, vancomycin MIC was associated with teicoplanin MIC (*P* < 0.001), daptomycin MIC (*P* < 0.001), but not with linezolid (*P* = 0.759).

**Table 1:** Molecular characteristics of methicillin-resistant *Staphylococcus aureus* (MRSA) isolates with or without reduced susceptibilities to anti-MRSA agents.

MIC	MIC of vancomycin (mg/L)			MIC of teicoplanin (mg/L)			MIC of daptomycin (mg/L)			MIC of linezolid (mg/L)		
Breakpoint, number (%)	≥1.5, n = 151 (79.9)	<1.5, n = 38 (20.1)	*P*	≥2, n = 163 (86.2)	<2, n = 26 (13.8)	*P*	≥0.38, n = 138 (73.0)	<0.38, n = 51 (27.0)	*P*	≥1.5, n = 121 (64.0)	<1.5, n = 68 (36.0)	*P*
**SCC*mec* type**												
I (n = 4)	2 (50)	2 (50)	0.181	2 (50)	2 (50)	0.092	3 (75)	1 (25)	1.000	3 (75)	1 (25)	1.000
II (n = 36)	27 (69.2)	9 (30.8)	0.128	36 (100)	0 (0)	0.005	32 (88.9)	4 (11.1)	0.021	22 (61.1)	14 (38.9)	0.546
III (n = 31)	29 (93.5)	2 (6.5)	0.048	29 (93.5)	2 (6.5)	0.261	29 (93.5)	2 (6.5)	0.004	13 (41.9)	18 (58.1)	0.005
IIIa (n = 34)	32 (94.1)	2 (5.9)	0.019	33 (94.3)	1 (5.7)	0.052	32 (91.4)	2 (8.6)	0.001	15 (42.9)	20 (57.1)	0.004
IIIb (n = 4)	4 (100)	0 (0)	0.585	4 (100)	0 (0)	1.000	3 (75.0)	1 (25.0)	1.000	1 (25.0)	3 (75.0)	0.100
IV (n = 61)	46 (75.4)	15 (24.6)	0.288	46 (75.4)	15 (24.6)	0.006	33 (54.1)	28 (45.9)	< 0.001	53 (86.9)	8 (13.1)	< 0.001
V (n = 2)	0 (0)	2 (100)	0.040	1 (50)	1 (50)	0.257	0 (0)	2 (100)	0.072	2 (100)	0 (0)	0.537
V_T_ (n = 17)	11 (64.7)	6 (35.3)	0.116	12 (70.6)	5 (29.4)	0.064	6 (35.3)	11 (64.7)	0.676	12 (70.6)	5 (29.4)	0.609
**HA-MRSA** (n = 109) **vs. CA-MRSA** (n = 80)	94 (86.2) vs. 57 (71.3)	15 (13.8) vs. 23 (28.7)	0.011	104 (95.4) vs. 59 (73.8)	5 (4.6) vs. 21 (26.2)	< 0.001	99 (90.8) vs. 39 (48.8)	10 (9.2) vs. 41 (51.2)	<0.001	54 (49.5) vs. 67 (83.8)	55 (50.5) vs. 13 (16.2)	<0.001
**HAI** (n = 120) **vs. CAI** (n = 69)	101 (84.2) vs. 50 (72.5)	19 (15.8) vs. 19 (27.5)	0.053	107 (89.2) vs. 56 (72.4)	13 (10.8) vs. 13 (27.6)	0.124	93 (77.5)vs. 45 (65.2)	27 (22.5) vs. 24 (34.8)	0.088	67 (55.8) vs. 54 (78.3)	53 (44.2) vs. 15 (78.3)	0.002
**Positive PVL** (n = 23)	14 (60.1)	9 (39.9)	0.024	17 (73.9)	6 (26.1)	0.099	10 (43.5)	13 (56.5)	< 0.001	18 (78.3)	5 (21.7)	0.166
**hVISA** (n = 14)	14 (100)	0 (0)	0.077	14 (100)	0 (0)	0.223	14 (100)	0 (0)	0.013	9 (64.3)	5 (35.7)	1.000

Note: MIC, minimum inhibitory concentration; SCC*mec*, staphylococcal cassette chromosome *mec*; HA-MRSA, hospital-associated methicillin-resistant *Staphylococcus aureus*, defined as an isolate carrying SCC*mec* type I, II, or III; CA-MRSA, community-associated methicillin-resistant *S*. *aureus*, defined as an isolate carrying SCC*mec* type IV or V; HAI, hospital-acquired infections; CAI, community-acquired infection; PVL, Panton-Valentine leucocidin; hVISA, vancomycin-heteroresistant *S*. *aureus*; vs., versus.

### Reduced susceptibility in MRSA with various SCC*mec* types

We found correlations of SCC*mec* types with MICs of antimicrobial agents (Table 1). In comparison with CA-MRSA (SCC*mec* type IV, V and V_T_), most HA-MRSA (SCC*mec* type I, II, III, IIIa and IIIb) showed higher incidence of vancomycin MIC ≥ 1.5 mg/L, teicoplanin MIC ≥ 2 mg/L, daptomycin MIC ≥ 0.38 mg/L, and linezolid < 1.5 mg/L (Table 1).

### Vancomycin-heteroresistant *S*. *aureus*

All of the 14 hVISA isolates (7.4%) belonged to HA-MRSA and showed significantly higher MIC for vancomycin (≥ 2 mg/L; *P* < 0.001), teicoplanin (≥ 6 mg/L; *P* < 0.001), and daptomycin (≥ 1 mg/L; *P* = 0.013) than non-hVISA isolates (Fig 1).

### Panton—Valentine leucocidin

PVL gene was present in 23 MRSA isolates (12.2%). All of these PVL-positive isolates belonged to CA-MRSA (Table 2).

**Table 2:** Panton-Valentine leucocidin genes and vancomycin heteroresistance in methicillin-resistant *Staphylococcus aureus* isolates with different staphylococcal cassette chromosome *mec* types causing hospital- or community-acquired infection.

Characteristics	PVL			hVISA			HAI/CAI		
Number (%)	PVL (+), n = 23 (12.2)	PVL (-), n = 166 (87.8)	*P*	hVISA, n = 14 (7.4)	Non-hVISA,n = 175 (92.6)	*P*	HAI, n = 120 (63.5)	CAI, n = 69 (36.5)	*P*
**SCC*mec* type**									
I (n = 4)	0 (0)	4 (100)	1.000	0 (0)	4 (100)	1.000	3 (75)	1 (25)	1.000
II (n = 36)	0 (0)	36 (100)	0.009	2 (5.6)	34 (94.4)	0.005	30 (83.3)	6 (16.7)	0.007
III (n = 31)	0 (0)	31 (100)	1.000	3 (9.7)	28 (90.3)	< 0.001	20 (64.5)	11 (35.5)	0.897
IIIa (n = 34)	0 (0)	34 (100)	1.000	9 (26.5)	25 (73.5)	< 0.001	26 (76.5)	8 (23.5)	0.115
IIIb (n = 4)	0 (0)	4 (100)	1.000	0 (0)	4 (100)	1.000	4 (100)	0 (0)	0.298
IV (n = 61)	6 (9.8)	55 (90.2)	< 0.001	0 (0)	61 (100)	1.000	29 (47.5)	32 (52.5)	0.002
V (n = 2)	1 (50)	1 (50)	0.122	0 (0)	2 (100)	1.000	0 (0)	2 (100)	0.132
V_T_ (n = 17)	16 (94.1)	1 (5.9)	< 0.001	0 (0)	17 (100)	1.000	8 (47.1)	9 (52.9)	0.186
**HA-MRSA** (n = 109) vs. **CA-MRSA** (n = 80)	0 (0) vs. 23 (28.8)	109 (100) vs. 57 (71.2)	< 0.001	14 (12.8) vs. 0 (0)	95 (87.2) vs. 80 (100)	< 0.001	83 (76.1) vs. 37 (46.2)	26 (23.9) vs. 43 (53.8)	< 0.001

Note: PVL, Panton-Valentine leucocidin; hVISA, vancomycin-heteroresistant *Staphylococcus aureus*; HAI, hospital-acquired infection; CAI, community-acquired infection; SCC*mec*, staphylococcal cassette chromosome *mec*; HA-MRSA, hospital-associated methicillin-resistant *S*. *aureus*; CA-MRSA, Community-associated methicillin-resistant *S*. *aureus*; vs., versus.

### Genotyping

The predominant ST in isolates of HA-MRSA is ST239 (66.3%) and that in CA-MRSA is ST59 (53.2%) as shown in supplemental information (S1 Fig and S2 Table).

### SCC*mec* types in HAI and CAI

More CA-MRSA (SCC*mec* type IV, V and V_T_) were isolated from CAI than HA-MRSA (SCC*mec* type I, II, III, IIIa and IIIb) (*P*< 0.001) and more HA-MRSA isolates were from HAI than CA-MRSA (*P*< 0.001) (Table 2). However, 37 (46.2%) CA-MRSA isolates from HAI and 26 (23.9%) HA-MRSA isolates from CAI (Table 2).

### Multi-stages risk factor analysis

Many factors correlated with mortality, including clinical severity (Pitt bacteraemia score, white blood cell count, C-reactive protein level), specific source (catheter-related infection), infection by specific SCC*mec* type (IIIA), HA-MRSA, or hVISA, infection by pathogen with higher vancomycin MIC (≥ 2 mg/L or ≥ 1.5 mg/L), teicoplanin MIC (≥ 6 mg/L, ≥ 4 mg/L, ≥ 3mg/L, ≥ 2mg/L), and daptomycin MIC (≥ 0.38 mg/L) and inappropriate initial therapy were associated with mortality (Table 3). After multivariate analysis, only Pitt bacteraemia score, C-reactive protein level, white blood cell count, catheter-related infection as infection source, infection by pathogen with vancomycin MIC ≥ 2 mg/L, infection by pathogen with vancomycin MIC ≥ 1.5 mg/L and inappropriate therapy were independent risk factors for 30-day mortality. Among these independent risk factors, infection by pathogen with vancomycin MIC ≥ 1.5 mg/L was the major risk factor (MRF1) for mortality with the highest odds ratio (adjusted odds ratio [AOR], 7.88; *P* = 0.010) (Table 3).

**Table 3:** Logistic regression analysis of risk factors for 30-day mortality.

Characteristics	Non-survivors	Survivors	Univariate analysis		Multivariate analysis^a^	
Number (%)	n = 55 (29.1)	n = 134 (70.9)	Odds ratio (95% CI)	*P*	Odds ratio (95% CI)	*P*
**Demographic data**						
Age, years	72 (55–81)	66 (49–78)	1.01 (1.00–1.03)	0.160		
Sex, male/female	30/25	74/60	0.97 (0.52–1.83)	0.932		
Length of stay in hospital	51 (43–57)	50 (33–57)	1.00 (0.98–1.01)	0.557		
**Underlying diseases**						
Charlson score	2 (1.5–3)	2 (1–3)	1.01 (0.88–1.17)	0.879		
Neoplastic disease	18 (32.7)	46 (34.3)	1.01 (0.86–1.18)	0.937		
Cardiac disease	13 (23.6)	36 (26.9)	0.60 (0.27–1.35)	0.218		
Cerebrovascular disease	12 (21.8)	28 (20.9)	1.12 (0.37–3.38)	0.843		
Diabetes	22 (40)	53 (40.0)	1.07 (0.56–2.05)	0.835		
Pulmonary disease	13 (23.6)	36 (26.9)	0,97 (0.41–1.98)	0.925		
Hepatic disease	11 (20)	27 (20.1)	1.53 (0.65–3.59)	0.329		
Renal disease	17 (30.9)	40 (29.9)	1.23(0.63–2.42)	0.551		
Peptic ulcer	5 (9.1)	13 (9.7)	0.93 (0.32–2.75)	0.897		
**Clinical severity**						
Pitt bacteraemia score	3 (2–4)	2 (1–3)	1.44 (1.20–1.74)	<0.001	1.41 (1.13–1.75)	0.002
White blood cell count, cells/nL	13.5 (8.6–20.5)	9.8 (6.8–13.4)	1.01 (0.99–1.04)	0.001	1.00 (1.00–1.00)	0.011
C-reactive protein level, mg/L	123 (79.6–166.2)	55.4 (34–134.7)	1.00 (1.00–1.01)	0.008	1.00 (1.00–1.01)	0.047
**Infection source**						
Catheter-related infection	18 (32.7)	23 (17.2)	3.39 (2.03–5.64)	<0.001	3.07 (1.16–8.07)	0.023
Pneumonia	12 (21.8)	27 (20.1)	1.11 (0.51–2.38)	0.797		
Primary bacteremia	7 (12.7)	30 (22.4)	0.51(0.21–1.23)	0.133		
Soft tissue infection	5 (9.1)	27 (20.1)	0.40 (0.14–1.09)	0.073		
Bone and joint infections	3 (5.5)	15 (11.2)	0.46 (0.13–1.65)	0.232		
Urinary tract infection	4 (7.3)	4 (3.0)	2.55 (0.61–10.58)	0.198		
Intra-abdominal infection	1 (1.8)	2 (1.5)	1.22 (0.11–13.76)	0.871		
**SCC*mec* type**						
I	2 (3.6)	2 (1.5)	2.49 (0.34–18.14)	0.368		
II	9 (16.4)	27 (20.1)	0.78 (0.34–1.78)	0.548		
III	9 (16.4)	22 (16.4)	1.00 (0.43–2.33)	0.993		
IIIa	18 (32.7)	16 (11.9)	3.59 (1.66–7.73)	0.001		
IIIb	1 (1.8)	3 (2.2)	0.81 (0.08–7.95)	0.855		
IV	13 (23.6)	48 (35.8)	0.56 (0.27–1.34)	0.106		
V	0 (0)	2 (1.5)	0.57 (0.02–15.67)	0.739		
V_T_	3 (5.5)	14 (10.4)	0.50 (0.14–1.79)	0.284		
HA-MRSA	39 (70.9)	70 (52.2)	2.23 (1.14–4.37)	0.020		
HAI	42 (76.4)	78 (58.2)	2.32 (1.14–4.72)	0.020		
PVL	4 (7.3)	19 (14.2)	0.48 (0.15–1.47)	0.195		
hVISA	9 (16.4)	5 (3.7)	5.05 (1.61–15.84)	0.006		
**MIC of vancomycin**						
MIC ≥3 mg/L	11 (20)	10 (7.5)	1.27 (0.78–2.06)	0.334		
MIC ≥2 mg/L	32 (58.2)	29 (21.6)	5.04 (2.56–9.90)	<0.001	3.49 (1.50–8.14)	0.004
MIC ≥1.5 mg/L	52 (94.5)	99 (73.9)	6.13 (1.80–20.88)	0.004	7.88 (1.62–38.28)	0.010
**MIC of teicoplanin**						
MIC ≥6 mg/L	11 (20)	7 (5.2)	4.54 (1.66–12.43)	0.003		
MIC ≥4 mg/L	28 (50.9)	39 (29.1)	2.53 (1.32–4.82)	0.005		
MIC ≥3 mg/L	42 (76.4)	77 (57.5)	2.39 (1.18–4.87)	0.016		
MIC ≥2 mg/L	54 (98.2)	109 (81.3)	12.39 (1.63–93.85)	0.015		
MIC ≥1.5 mg/L	55 (100)	125 (93.3)	8.60 (0.44–167.04)	0.155		
**MIC of daptomycin**						
MIC ≥1.5 mg/L	4 (7.3)	9 (6.7)	1.09 (0.32–3.70)	0.891		
MIC ≥1 mg/L	13 (23.6)	19 (14.2)	1.87 (0.85–4.12)	0.119		
MIC ≥0.38 mg/L	46 (83.6)	92 (68.7)	2.33 (1.05–5.20)	0.038		
**MIC of linezolid**						
MIC ≥2 mg/L	6 (10.9)	24 (17.9)	0.56 (0.22–1.46)	0.236		
MIC ≥1.5 mg/L	35 (63.6)	86 (64.2)	0.98 (0.51–1.88)	0.944		
MIC ≥1 mg/L	55 (100)	125 (93.3)	8.60 (0.44–167.04)	0.155		
**Inappropriate initial therapy**	24 (43.7)	17 (12.7)	9.91 (5.66–17.36)	<0.001	6.78 (2.61–17.60)	<0.001

Note: Data are presented as median value (interquartile range: Q1-Q3) for continuous variables and number of cases (%) for categorical variables. SCC*mec*, staphylococcal cassette chromosome *mec*; HA-MRSA, hospital-associated methicillin-resistant *Staphylococcus aureus*, defined as an isolate possessing SCC*mec* type I, II, or III; HAI, hospital-acquired infections; PVL, Panton-Valentine leucocidin; hVISA, vancomycin-heteroresistant *S*. *aureus*; MIC, minimum inhibitory concentration.

^a^All variables with a *P* value < 0.20 in the univariable analysis were considered for inclusion in the logistic regression model in the multivariable analysis. A forward stepwise selection process was utilized. It was found that only Pitt bacteraemia score, C-reactive protein level, white blood cell count, catheter-related infection as infection source, infection by MRSA with vancomycin MIC ≥ 2 μg/mL, infection by MRSA with vancomycin MIC ≥ 1.5 μg/mL and inappropriate therapy were statistically significant risk factors for 30-day mortality.

After multivariate analysis, infection by pathogen with daptomycin MIC ≥ 0.38 mg/L was the major independent risk factor (MRF2) associated with vancomycin MIC ≥ 1.5 mg/L (AOR, 2.82; 95% confidence interval [CI], 1.38–5.78; *P* = 0.005).

After multivariate analysis, infection by pathogen belonging to HA-MRSA was the major independent risk factor (MRF3) associated with infection by pathogen with daptomycin MIC ≥ 0.38 mg/L (AOR, 8.06; 95% CI, 3.59–18.11; *P* < 0.001), followed by infection by pathogen with teicoplanin MIC ≥ 2 mg/L (AOR 5.08; 95% CI, 1.86–13.88; *P* = 0.002).

After multivariate analysis, prior use of broad-spectrum antibiotics, including vancomycin, teicoplanin, carbapenems, extended-spectrum cephalosporins, fluoroquinolones or piperacillin/ tazobactam, for at least 5 days (MRF4) was the major independent risk factor associated with infection by pathogen belonging to HA-MRSA (AOR, 108.77; 95% CI, 14.14–836.64; *P* < 0.001), followed by age (AOR, 1.02; 95% CI, 1.003–1.041; *P* = 0.023). Infection by pathogen with linezolid MIC ≥ 1.5 mg/L was also an independent risk factor with the lowest odds ratio (AOR, 0.265; 95% CI, 0.11–0.66; *P* = 0.005).

Before the occurrence of bacteremia, a total of 71 patients had received broad-spectrum antibiotics for treating catheter-related infections (n = 32, 45%), pneumonia (n = 21, 29.6%), soft tissue infection (n = 8, 11.3%), bone and joint infections (n = 6, 8.5%), urinary tract infection (n = 3, 4.2%), and intra-abdominal infection (n = 1, 1.4%). Catheter-related infections included Hickmen, Port-A, central venous catheter and shunt infections.

### Backward root analysis for mortality

**Fig 2:**
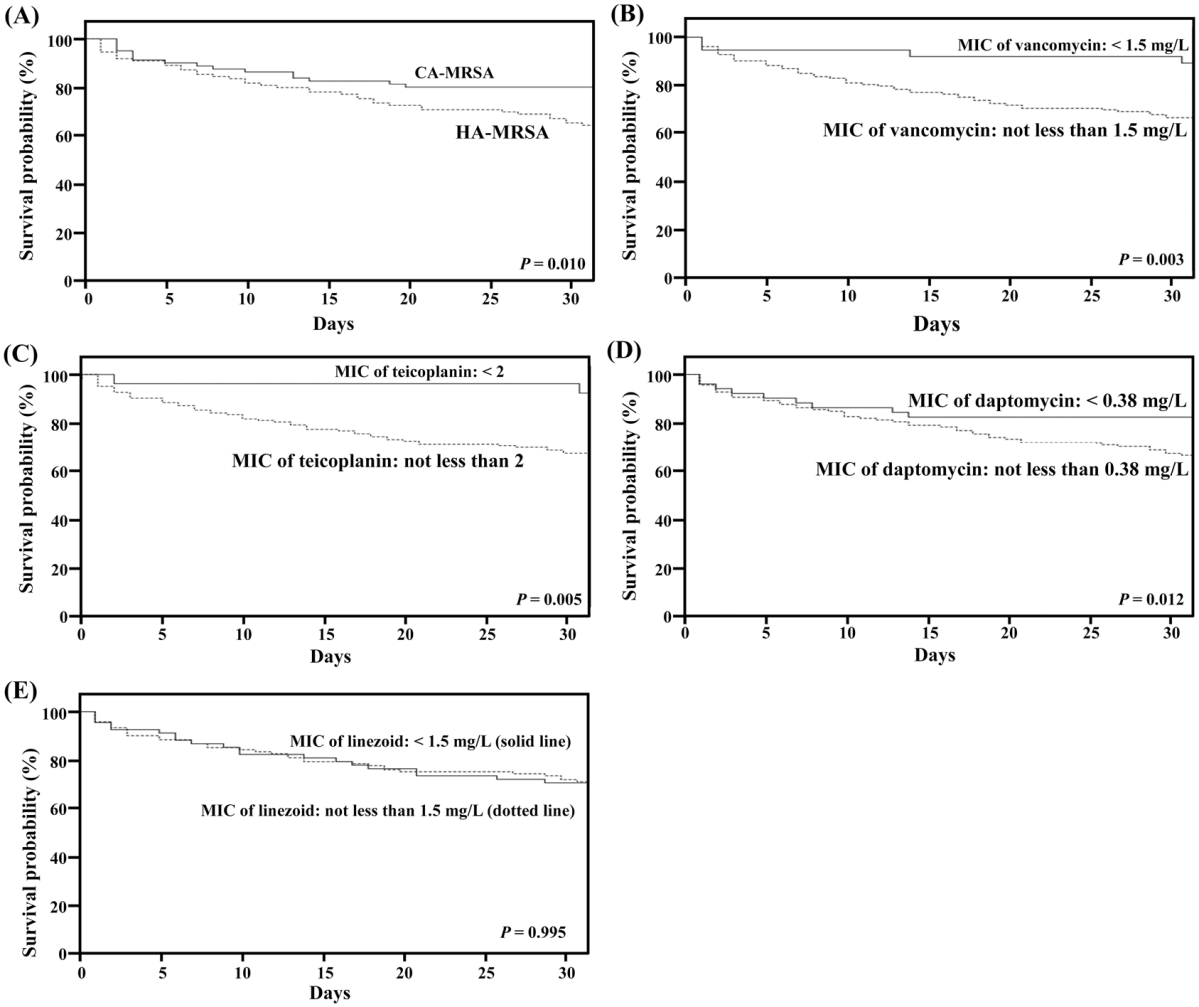
The Kaplan-Meier estimate of survival curves of patients with methicillin-resistant *Staphylococcus aureus* (MRSA) bacteremia. The Kaplan-Meier survival curve of patients infected by community-associated MRSA (solid line) is compared with those by hospital-associated MRSA (dotted line) (*P* < 0.010, by log-rank test) (A). The Kaplan-Meier survival curve of patients infected by MRSA with vancomycin minimum inhibitory concentration (MIC) ≥ 1.5 mg/L (dotted line) is compared with those by MRSA with vancomycin MIC < 1.5 mg/L (solid line) (*P* = 0.003, by log-rank test) (B). The Kaplan-Meier survival curve of patients infected by MRSA with teicoplanin MIC ≥ 2 mg/L (dotted line) is compared with those by MRSA with teicoplanin MIC < 2 mg/L (solid line) (*P* = 0.005, by log-rank test) (C). The Kaplan-Meier survival curve of patients infected by MRSA with daptomycin MIC ≥ 0.38 mg/L (dotted line) is compared with those by MRSA with daptomycin MIC < 0.38 mg/L (solid line) (*P* = 0.012, by log-rank test) (D). The Kaplan-Meier survival curve of patients infected by MRSA with linezolid MIC ≥ 1.5 mg/L (solid line) is compared with those by MRSA with linezolid MIC < 1.5 mg/L (dotted line) (*P* < 0.995, by log-rank test) (E).

After the above multi-stages risk factor analysis, we were able to summarize a development stages for mortality due to MRSA bacteremia (Table 4). A prior infection, such as catheter-related infections, pneumonia, or soft tissue infection, was stage 1. Broad-spectrum antibiotic use for treating one of these prior infections was stage 2. From stage 3 to stage 5, HA-MRSA with higher vancomycin MIC, higher teicoplanin MIC, and higher daptomycin MIC (Table 1) was selected from broad-spectrum antibiotic pressure from stage 2. After analysis by log-rank test, higher incidence of mortality correlated with MRSA with higher vancomycin MIC (*P* = 0.003), higher teicoplanin MIC (*P* = 0.005), and higher daptomycin MIC (*P* = 0.012) (Fig 2). Therefore, infection by HA-MRSA was associated with high mortality (*P* = 0.010 by log-rank test).

**Table 4:** Backward root analysis for main risk factor at different stages from the development of methicillin-resistant *Staphylococcus aureus* bacteremia to mortality and proposed solution for each stage.

Stages	Stage 1	Stage 2	Stage 3	Stage 4	Stage 5	Stage 6
MRF or outcome	Catheter-related infections, pneumonia, or soft tissue infection (MRF5)	Prior exposure to broad spectrum antibiotics ≥5 days (MRF4)	HA-MRSA (MRF3)	Infection by MRSA with daptomycin MIC ≥0.38 mg/L (MRF2)	Infection by MRSA with vancomycin MIC ≥1.5 mg/L (MRF1)	Mortality due to MRSA bacteremia
Recommended intervention	Early detection of HA-MRSA and removal of catheter	Encouraging prudent antibiotic use	Detection of HA-MRSA bacteremia	Linezoid recommended for therapy	Linezoid recommended for therapy	

Note: MRF, main risk factor; HA-MRSA, hospital-associated methicillin-resistant *Staphylococcus aureus*, defined as an isolate possessing SCC*mec* type I, II, or III.

## Discussion

At least three important findings in MRSA bacteremia were reported in this study. Firstly, in contrast to CA-MRSA (53.2% ST59), HA-MRSA (66.3% ST239) was found associated with increased vancomycin MIC, teicoplanin MIC, and daptomycin MIC. HA-MRSA tended to simultaneously express reduced susceptibility to most anti-MRSA agents, except linezolid. Secondly, although patients infected by MRSA with vancomycin MIC ≥ 1.5 mg/L or teicoplanin MIC > 1.5 mg/L have been proved to have a higher mortality rate [3,6], we further found in this study that bacteremic patients infected by MRSA with daptomycin MIC ≥ 0.38 mg/L also showed a higher mortality. Thirdly, by backward root analysis, there were multiple stages from the source of infection to mortality due to MRSA bacteremia. MRSA with vancomycin MIC ≥ 1.5 mg/L and inappropriate initial therapy were the two most important risk factors for mortality. Risk factors, including HA-MRSA, MRSA with vancomycin MIC ≥ 1.5 mg/L, teicoplanin MIC ≥ 1.5 mg/L, and daptomycin MIC ≥ 0.38 mg/L, were associated with each other and the combined effect is a higher risk of mortality in the bacteremic patients.

Similar to previous reports, we found in this study that catheter-related infections, pneumonia, and soft tissue infections usually preceded MRSA bacteremia [6]. Broad-spectrum antibiotics were prescribed for the empirical treatment of such infections. This therefore generated a high antibiotic selective pressure for subsequent breakthrough infection caused by drug-resistant organisms, such as MRSA with a higher antimicrobial MIC. Likely in previous studies, use of broad-spectrum antibiotics such as fluoroquinolones has been found in association with increased rates of MRSA acquisition [24].

We found that HA-MRSA with higher MIC of anti-MRSA agents were selected by the prior use of broad-spectrum antibiotics to cause subsequent bacteremia. The association of genotypes of MRSA with increased vancomycin MIC was examined in previous studies [8,9]. A significant association between SCC*mec* II/III and elevated vancomycin MIC was reported [8]. Higher vancomycin MICs were also linked to specific clonal complexes (CCs) and HA-MRSA [10,11]. CC8 was associated with elevated vancomycin MIC, and in contrast, low vancomycin MIC with CC22, CC88, and CC188 [9]. SCC*mec* is a mobile element that was spread through horizontal gene transfer. We observed that HA-MRSA with increased MIC of anti-MRSA drugs (except linezolid) was also associated with higher mortality in this study, suggesting that other intrinsic microbial factors were involved in the pathogenesis of MRSA bacteremia with a high mortality. In fact, in a previous study, in comparison to patients with CA-MRSA infections, the higher risk for treatment failure among patients with HA-MRSA infections suggested that HA-MRSA could possess an intrinsic strain-specific virulence factor [10].

Before 2006, MRSA isolates were considered susceptible to vancomycin when MIC was ≤ 4 mg/mL; however, this breakpoint was decreased to ≤ 2 mg/L due to poor therapeutic results in patients with infections caused by MRSA with MIC > 2 mg/L [25]. Thereafter, elevated vancomycin MIC (≥ 1.5 mg/L in MRSA and > 1.5 mg/L in methicillin-susceptible *S*. *aureus* [MSSA]) has been proved an independent risk factor for 30-day mortality of patients with *S*. *aureus* bacteremia, regardless of resistance to methicillin or the treatment administered [4,26]. Given the results, and others, not only vancomycin MIC but also teicoplanin MIC and daptomycin MIC could be considered surrogate markers for pathogen-specific factors responsible for worse outcomes or increased virulence secondary to antibiotic resistance [27]. Although vancomycin remains to be the first-line therapy for severe MRSA infections [28], there are now sufficient data demonstrating the efficacy of daptomycin and linezolid for both MSSA and MRSA infections [29,30]. For the treatment of MRSA bacteremia, only daptomycin and vancomycin have been approved by Food and Drug Administration (FDA), but therapy with linezolid, an alternative antibiotic, showed outcomes non-inferior to vancomycin treatment in patients [30]. Infection caused by MRSA with higher vancomycin MIC, which showed thicker cell wall, has been reported associated with higher risk of daptomycin treatment failure. This is a phenomenon that can be explained by the inability of daptomycin, molecular weight of which is 4.8 times larger than linezolid, to diffuse to its active site through a thickened cell wall [29]. Indeed, our results showed a positive correlation between vancomycin MIC and daptomycin MIC in MRSA. On the other hand, our results revealed that the linezolid MIC did not increase along with the creeping vancomycin MIC in MRSA. Therefore, therapy with linezolid may be better than that with daptomycin for infection caused by MRSA with higher vancomycin MIC.

In this study, inappropriate initial therapy was found the second most important risk factor for mortality. This was also described in previous studies that identified delay in the initiation of appropriate antimicrobial therapy as an integral determinant of poor clinical outcomes for severe diseases, such as MRSA bacteremia [12,13]. Based on this, in case of persistent MRSA bacteremia following initial treatment, the 7-day threshold to seeking alternative combination antibiotic therapy is recommended to be shortened to 3–4 days [30].

In summary, MIC of vancomycin, teicoplanin and daptomycin must be checked in 2 days for choosing an effective initial antibiotic therapy for patients with MRSA bacteremia. If a patient shows a poor response to vancomycin and only vancomycin MIC is known, the study suggests that therapy with linezolid might lead to a better outcome for MRSA isolates with vancomycin MIC ≥ 1.5 mg/L. Such isolates usually showed a reduced susceptibility to daptomycin. Giving an effective anti-MRSA agent without an MIC indicating reduced susceptibility in 2 days is crucial for reducing mortality of MRSA bacteremia.

## Supporting Information

S1 Fig Sequence types by multilocus sequence typing of isolates causing hospital-acquired infection (A) and community-acquired infection (B). (TIF)

S1 Table Clinical and laboratory standards institute breakpoints (mg/L) for methicillin-resistant *Staphylococcus aureus*. (DOCX)

S2 Table Sequence types by multilocus sequence typing identified among isolates with different staphylococcal cassette chromosome *mec* types with or without Panton-Valentine leucocidins. (DOCX)
