# Supplementary figures and images for: Impact of Molecular Epidemiology and Reduced Susceptibility to Glycopeptides and Daptomycin on Outcomes of Patients with Methicillin-Resistant Staphylococcus aureus Bacteremia
Source: PLoS One. 2015 Aug 21;10(8):e0136171. doi: 10.1371/journal.pone.0136171 (PMC4546585; doi:10.1371/journal.pone.0136171)

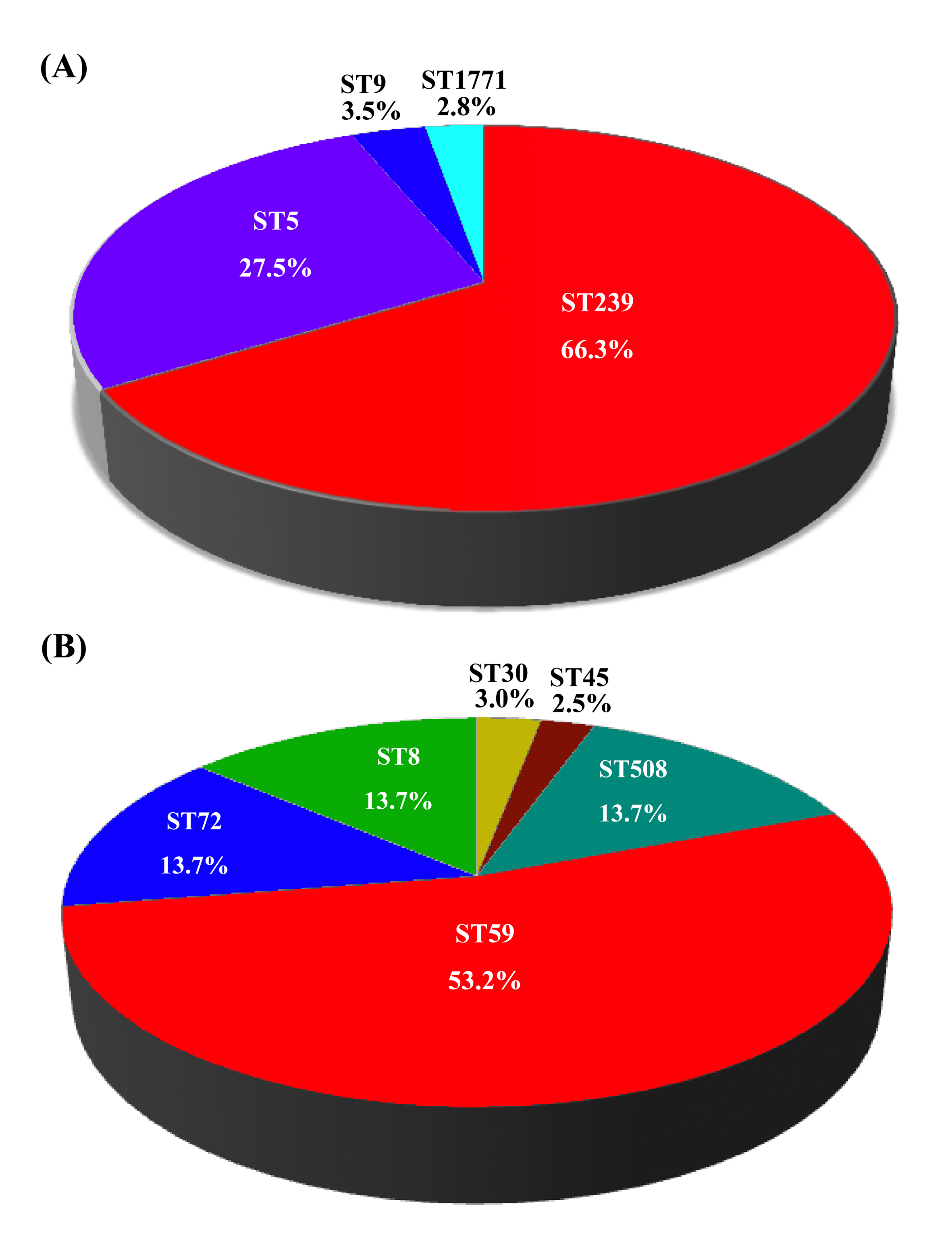

Supplement: S1 Fig — (TIF) [file pone.0136171.s001.tif]
