# Supplementary material for: Impact of Molecular Epidemiology and Reduced Susceptibility to Glycopeptides and Daptomycin on Outcomes of Patients with Methicillin-Resistant Staphylococcus aureus Bacteremia
Source: PLoS One. 2015 Aug 21;10(8):e0136171. doi: 10.1371/journal.pone.0136171 (PMC4546585; doi:10.1371/journal.pone.0136171)
Supplement: S1 Table — (DOCX) [file pone.0136171.s002.docx]

**Table S1. Clinical and laboratory standards institute breakpoints (mg/L) for methicillin-resistant *Staphylococcus aureus*.**

| **Anti-MRSA Agents** | **Susceptible** | **Intermediately Resistant** | **Resistant** |
| --- | --- | --- | --- |
| Vancomycin | ≦2 | 4~8 | ≧16 |
| Teicoplanin | ≦8 | 16 | ≧32 |
| Linezoid | ≦4 | - | ≧8 |
| Daptomycin | ≦1 | - | - |
