# Supplementary material for: Impact of Molecular Epidemiology and Reduced Susceptibility to Glycopeptides and Daptomycin on Outcomes of Patients with Methicillin-Resistant Staphylococcus aureus Bacteremia
Source: PLoS One. 2015 Aug 21;10(8):e0136171. doi: 10.1371/journal.pone.0136171 (PMC4546585; doi:10.1371/journal.pone.0136171)
Supplement: S2 Table — (DOCX) [file pone.0136171.s003.docx]

**Table S2. Sequence types by multilocus sequence typing identified among isolates with different staphylococcal cassette chromosome *mec* types with or without Panton-Valentine leucocidins**

| **SCC*mec* Type** | **PVL** | **No. of ST/Total No. Tested** | **STs Distribution** | **Predicted %** |
| --- | --- | --- | --- | --- |
| I (n=4) | negative (n=4) | 1/1 | ST239 | 2.11 |
| II (n=36) | negative (n=36) | 10/12 | ST5 | 15.87 |
|  |  | 1/12 | ST239 | 1.59 |
|  |  | 1/12 | ST1771 | 1.59 |
| III (n=31) | negative (n=31) | 10/10 | ST239 | 16.40 |
| IIIa (n=34) | negative (n=34) | 8/9 | ST239 | 15.99 |
|  |  | 1/9 | ST9 | 2.00 |
| IIIb (n=4) | negative (n=4) | 4/4 | ST239 | 2.11 |
| IV (n=61) | positive (n=6) | 3/5 | ST59 | 1.90 |
|  |  | 2/3 | ST30 | 1.27 |
|  | negative (n=55) | 2/5 | ST59 | 11.64 |
|  |  | 1/5 | ST8 | 5.82 |
|  |  | 1/5 | ST72 | 5.82 |
|  |  | 1/5 | ST508 | 5.82 |
| V (n=2) | positive (n=1) | 1/1 | ST59 | 0.53 |
|  | negative (n=1) | 1/1 | ST45 | 0.53 |
| V_T_ (n=17) | positive (n=16) | 16/16 | ST59 | 8.47 |
|  | negative (n=1) | 1/1 | ST45 | 0.53 |

Abbreviations: SCC*mec,* staphylococcal cassette chromosome *mec*; PVL, Panton-Valentine leucocidin; No., number; ST, sequence type.
